# Plasma Sphingosine-1-Phosphate Levels Are Associated with Progression of Estrogen Receptor-Positive Breast Cancer

**DOI:** 10.3390/ijms222413367

**Published:** 2021-12-13

**Authors:** Mayuko Ikarashi, Junko Tsuchida, Masayuki Nagahashi, Shiho Takeuchi, Kazuki Moro, Chie Toshikawa, Shun Abe, Hiroshi Ichikawa, Yoshifumi Shimada, Jun Sakata, Yu Koyama, Nobuaki Sato, Nitai C. Hait, Yiwei Ling, Shujiro Okuda, Kazuaki Takabe, Toshifumi Wakai

**Affiliations:** 1 Division of Digestive and General Surgery, Niigata University Graduate School of Medical and Dental Sciences, Niigata 951-8510, Japan; m-ikarashi@niigata-cc.jp (M.I.); j-tsuchida@med.niigata-u.ac.jp (J.T.); kmoro@med.niigata-u.ac.jp (K.M.); ctoshikawa@med.niigata-u.ac.jp (C.T.); s-abe@med.niigata-u.ac.jp (S.A.); hichikawa-nii@med.niigata-u.ac.jp (H.I.); shimaday@med.niigata-u.ac.jp (Y.S.); jsakata2@med.niigata-u.ac.jp (J.S.); kazuaki.takabe@roswellpark.org (K.T.); wakait@med.niigata-u.ac.jp (T.W.); 2 Department of Breast Oncology, Niigata Cancer Center Hospital, Niigata 951-8566, Japan; nobus@niigata-cc.jp; 3 Department of Surgery, Division of Breast and Endocrine Surgery, Hyogo College of Medicine, Nishinomiya 663-8501, Japan; 4 Division of Bioinformatics, Niigata University Graduate School of Medical and Dental Sciences, Niigata 951-8510, Japan; shiho-takeuchi@med.niigata-u.ac.jp (S.T.); seraphwyl@med.niigata-u.ac.jp (Y.L.); okd@med.niigata-u.ac.jp (S.O.); 5 Department of Nursing, Graduate School of Health Sciences, Niigata University, Niigata 951-8518, Japan; yukmy@clg.niigata-u.ac.jp; 6 Department of Molecular and Cellular Biology, Roswell Park Comprehensive Cancer Center, Elm & Carlton Streets, Buffalo, NY 14263, USA; nitai.hait@roswellpark.org; 7 Breast Surgery, Department of Surgical Oncology, Roswell Park Comprehensive Cancer Center, Elm & Carlton Streets, Buffalo, NY 14263, USA; 8 Department of Surgery, Jacobs School of Medicine and Biomedical Sciences, University at Buffalo, The State University of New York, Buffalo, NY 14260, USA

**Keywords:** sphingosine-1-phosphate, breast cancer, lymph node metastasis, plasma, mass spectrometry, estrogen receptor, progesterone receptor, hormone therapy

## Abstract

Although numerous experiments revealed an essential role of a lipid mediator, sphingosine-1-phosphate (S1P), in breast cancer (BC) progression, the clinical significance of S1P remains unclear due to the difficulty of measuring lipids in patients. The aim of this study was to determine the plasma concentration of S1P in estrogen receptor (ER)-positive BC patients, as well as to investigate its clinical significance. We further explored the possibility of a treatment strategy targeting S1P in ER-positive BC patients by examining the effect of FTY720, a functional antagonist of S1P receptors, on hormone therapy-resistant cells. Plasma S1P levels were significantly higher in patients negative for progesterone receptor (PgR) expression than in those positive for expression (*p* = 0.003). Plasma S1P levels were also significantly higher in patients with larger tumor size (*p* = 0.012), lymph node metastasis (*p* = 0.014), and advanced cancer stage (*p* = 0.003), suggesting that higher levels of plasma S1P are associated with cancer progression. FTY720 suppressed the viability of not only wildtype MCF-7 cells, but also hormone therapy-resistant MCF-7 cells. Targeting S1P signaling in ER-positive BC appears to be a possible new treatment strategy, even for hormone therapy-resistant patients.

## 1. Introduction

Worldwide, breast cancer (BC) is one of the most prevalent malignant neoplasms in women according to epidemiological data [1]. Despite advances in multidisciplinary treatment strategies, which include surgery; radiation therapy; hormonal therapy; chemotherapy; and, most recently, immunotherapy, metastatic BC remains incurable [2,3]. Elucidating mechanisms underlying BC progression and metastasis, and finding therapeutic targets, are the keys to developing new strategies for cure [4].

Recently, increasing evidence has revealed lipid mediators are important players in progression and metastasis of various cancers including BC [5,6,7,8,9,10,11,12]. Among them all, sphingosine-1-phosphate (S1P) is one of the most important bioactive lipid mediators, which promotes cancer proliferation, invasion, and metastasis by affecting the cancer itself and its microenvironment [13,14,15,16,17,18,19,20,21,22,23,24,25,26]. S1P is produced from sphingosine (Sph) intracellularly by two different sphingosine kinases, SphK1 and SphK2. Intracellular S1P is transported by several transporters, including two ATP-binding cassette (ABC) transporters, ABCC1 and ABCG2, to the outside of cells [27,28,29,30]. There, S1P binds to five S1P-specific G protein-coupled receptors, S1PR1–5, on the cell membrane in an autocrine and paracrine manner and regulates many cellular functions [6,31,32,33]. Although experimental data, including ours, revealed essential roles of S1P in BC progression, there has been a paucity of data on the clinical significance of S1P due to the difficulty of measuring lipids in patients. Utilizing mass spectrometry, we recently demonstrated that the S1P concentration in tumor tissue is higher than in normal breast tissue, and higher S1P production in the tumor is related to a high frequency of lymph node metastasis in BC patients [34,35]. However, systemic plasma levels of S1P in BC progression in patients have not been investigated to date.

On the basis of previous basic research showing that S1P plays a pivotal role in estrogen-mediated signaling pathways in BC [28,36,37,38,39,40], we hypothesized that plasma S1P levels increase as the cancer progresses in estrogen receptor (ER)-positive BC patients. The aim of the current study was to determine the concentration of bioactive sphingolipids, including S1P, in plasma of patients with ER-positive BC using high-sensitivity mass spectrometry, as well as to investigate associations between plasma S1P concentrations and pathological findings related to cancer progression. We further suppressed S1P signaling in vitro to explore the possibility of a treatment strategy that targets S1P signaling in ER-positive BC patients.

## 2. Results

### 2.1. Plasma S1P Levels Were Higher in Advanced BC Patients

The levels of sphingolipids, including S1P, dihydro-S1P (DHS1P), Sph, and dihydro-Sph (DHSph), were determined in plasma samples from 126 ER-positive BC patients. DHS1P is an analogue of S1P and another lipid mediator, which is produced from DHSph by sphingosine kinases. Clinicopathologic characteristics of the 126 BC patients are summarized in Table 1.

**Figure 1:**
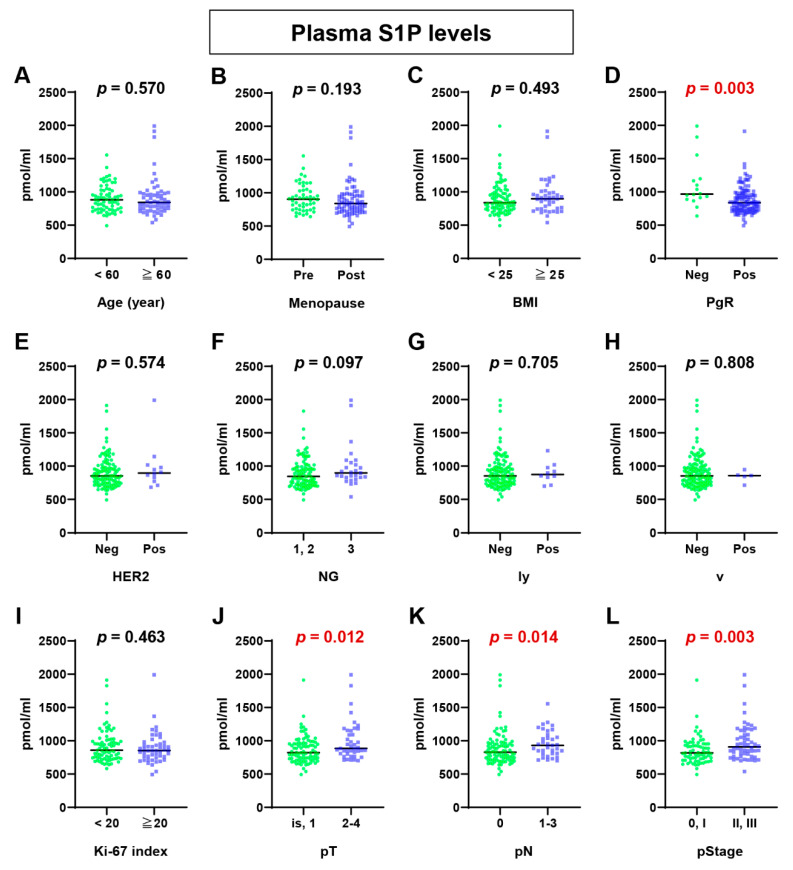
Sphingosine-1-phosphate (S1P) levels in plasma of estrogen receptor-positive breast cancer patients. S1P levels were determined by mass spectrometry and compared between patients of <60 years and ≥60 years (**A**); premenopause (Pre) and postmenopause (Post) (**B**); body mass index (BMI) <25 or ≥25 (**C**); negative (Neg) and positive (Pos) for progesterone receptor (PgR) (**D**); negative and positive for HER2 expression/amplification (**E**); NSAS nuclear grade (NG) 1, 2, or 3 (**F**); negative and positive for lymphatic invasion (ly) (**G**); negative and positive for vascular invasion (v) (**H**); Ki-67 labeling index <20 and ≥20 (**I**); pathological primary tumor (pT) 1 and 2–4 (**J**); pathological regional lymph node (pN) 0 and 1–3 (**K**); pathological stage (pStage) 0, I, II, and III (**L**). Horizontal lines indicate median values. The Mann–Whitney *U* test was performed for statistical analysis, in which all tests were two-sided, and *p*-values < 0.05 were considered statistically significant (shown in red color).

First of all, plasma S1P levels in ER-positive BC patients were analyzed on the basis of each clinicopathological factor (Figure 1). We found that plasma S1P levels were significantly higher in patients negative for progesterone receptor (PgR) expression than those positive for PgR expression (*p* = 0.003, Figure 1D). Plasma S1P levels were also significantly higher in patients with larger tumor size (higher pT; *p* = 0.012, Figure 1J), lymph node metastasis (higher pN; *p* = 0.014, Figure 1K), and advanced cancer stage (higher pStage; *p* = 0.003, Figure 1L). The plasma S1P levels tended to be higher in patients with higher nuclear grade (*p* = 0.097). Taken together, it appears that the plasma S1P levels were associated with tumor progression, in addition to the PgR status (Figure 1).

Second of all, plasma levels of DHS1P, which is an analogue of S1P and is produced from DHSph by sphingosine kinases, were analyzed on the basis of the same clinicopathological factors as those shown in Figure 1. The plasma levels of DHS1P showed a similar tendency as the plasma S1P levels (Table 2, Figure S1). In particular, plasma DHS1P levels were significantly higher in patients with higher nuclear grade (Table 2, Figure S1). Plasma DHS1P levels were also significantly higher in patients with higher pStage. Plasma DHS1P levels in patients negative for PgR expression and with higher pT tended to be higher compared to those positive for PgR expression and with lower pT (*p* = 0.069 and *p* = 0.066, respectively).

S1P and DHS1P have been reported to function as cancer-promoting lipid mediators. On the other hand, Sph and DHSph act as mediators that promote apoptosis. Therefore, it is of interest to investigate the plasma levels of Sph (Table 2, Figure S2) and DHSph (Table 2, Figure S3) in BC patients. We found that plasma Sph levels were significantly higher in patients with higher pN (*p* = 0.024) and with higher pStage (*p* = 0.037; Table 2, Figure S2). Interestingly, postmenopausal patients tended to have lower plasma Sph levels than premenopausal patients (*p* = 0.051, Table 2, Figure S2). DHSph levels only differed significantly between patients with positive for PgR expression and those negative for PgR expression (*p* = 0.026).

### 2.2. Possibility of Suppression of S1P Signaling in ER-Positive BC

**Figure 2:**
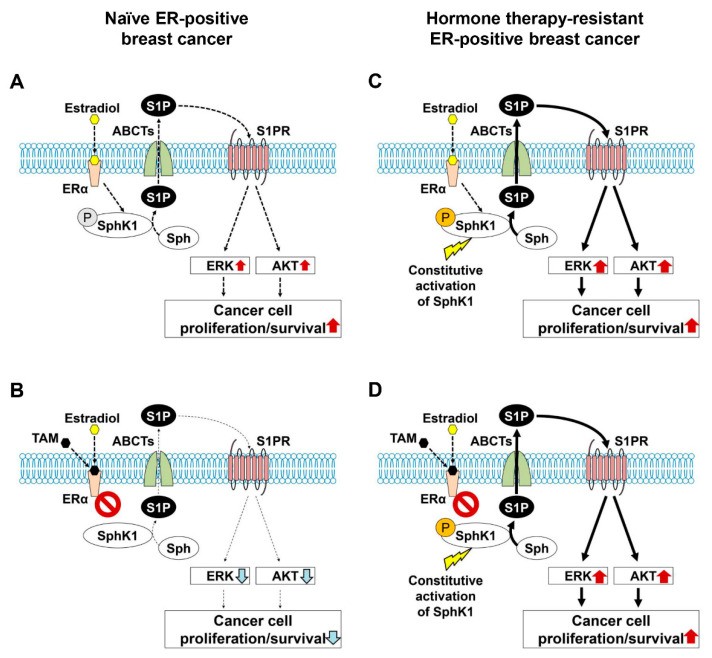
Scheme highlighting the importance of sphingosine-1-phosphate (S1P) signaling in breast cancer (BC) with estrogen receptor (ER)-positive status, even with hormone therapy resistance. Binding of estradiol to ERα stimulates production and release of S1P via ABC transporters (ABCTs) ABCC1 and ABCG2. Outside of the cells, S1P binds to S1P receptors to stimulate ERK and AKT, leading to activation of further downstream signaling pathways important for BC cell proliferation and survival (**A**). Tamoxifen (TAM), an ERα antagonist, blocks production and release of S1P, so that BC progression may be suppressed (**B**). In contrast, production and release of S1P by constitutive activation of SphK1 may result in an enhancement of cell proliferation and survival without estradiol (**C**). Hormone therapies such as TAM are unlikely to be effective in a situation of high S1P with constitutive activation of SphK1 in ER-positive BC (**D**).

Our data revealed that plasma S1P levels are associated with progression of ER-positive BC in patients. This finding is consistent with previous experimental data that revealed S1P plays an important role in the nongenomic signaling pathway of ER-positive BC [28,40,42]. In previous studies, it was demonstrated that SphK1, but not SphK2, is involved in S1P export from BC cells through the action of ABCC1 and ABCG2, which plays a part of signal transduction for the non-genomic action of estradiol and tamoxifen resistance in BC (Figure 2A–C) [27,28,29]. Recently, it has been reported that SphK1 activation by estrogen receptor α36 contributes to tamoxifen resistance in breast cancer [43]. It was suggested that S1P is critical not only in the progression of ER-positive breast cancer, but also in the development of tamoxifen resistance in those patients [43]. On the basis of these previous findings, we hypothesized that S1P contributes to resistance to hormone therapy and that blocking S1P signaling would improve the susceptibility of hormone therapy-resistant BC (Figure 2D). Therefore, we explored the clinical significance of suppression of S1P signaling in ER-positive BC patients.

**Figure 3:**
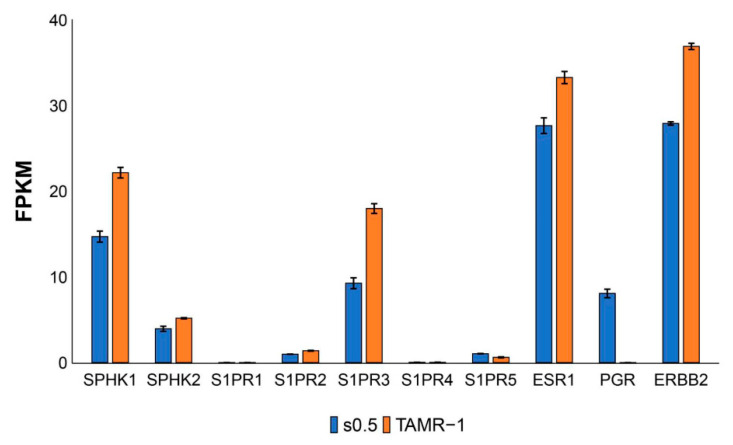
Expression levels of S1P-related genes, ESR1, PGR, and HER2, on wildtype MCF-7 cells (MCF-7/S0.5) and tamoxifen-resistant MCF-7 cells (MCF-7/TAMR-1). Expression levels of S1P-related genes, ESR1, PGR, and HER2, were assessed by RNA-seq. Data are shown as means ± SE.

**Figure 4:**
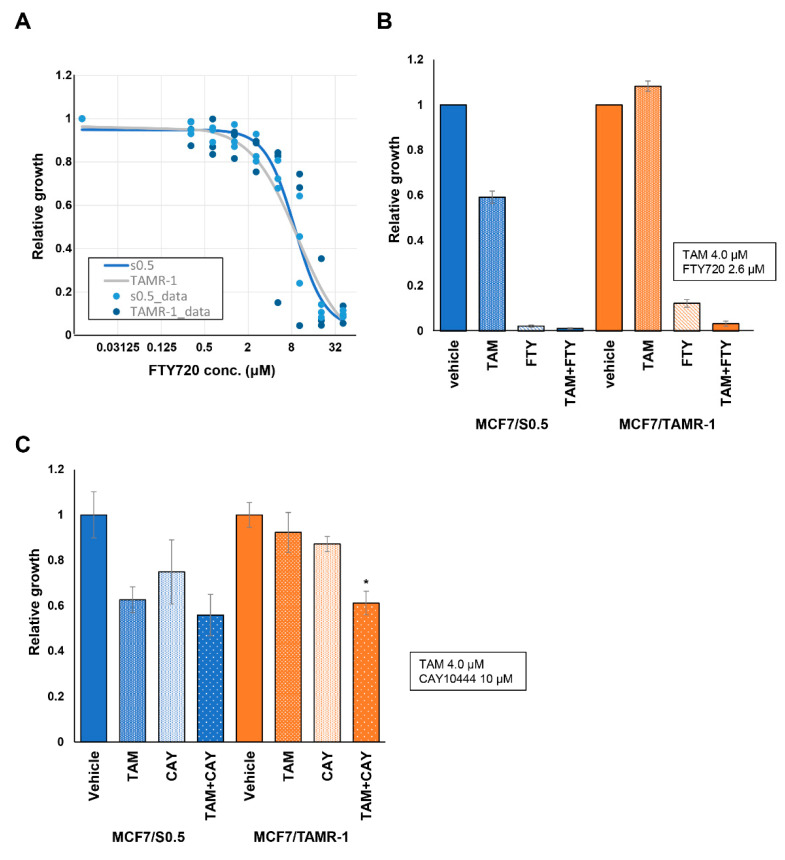
Effect of FTY720 on the estrogen receptor (ER)-positive breast cancer cell line, MCF-7, with or without hormone therapy resistance. Effects of different concentrations of FTY720 on the viability of wildtype MCF-7 cells (MCF-7/S0.5) and tamoxifen-resistant MCF-7 cells (MCF-7/TAMR-1) were assessed using WST-8 assay (**A**). The effect of tamoxifen (TAM), FTY720, and both TAM and FTY720 on MCF-7/S0.5 and MCF-7/TAMR-1 were assessed (**B**). The effects of tamoxifen (TAM), and a selective antagonist for S1PR3, CAY10444 (CAY), on MCF-7/S0.5 and MCF-7/TAMR-1 growth were assessed individually and in combination (* *p* = 0.0017 vs. TAM, *p* = 0.012 vs. CAY for MCF-7/TAMR-1) (**C**). FPKM, fragments per kilobase of transcript per million mapped reads; TAM, tamoxifen; FTY, FTY720; CAY, CAY10444.

**Figure 5:**
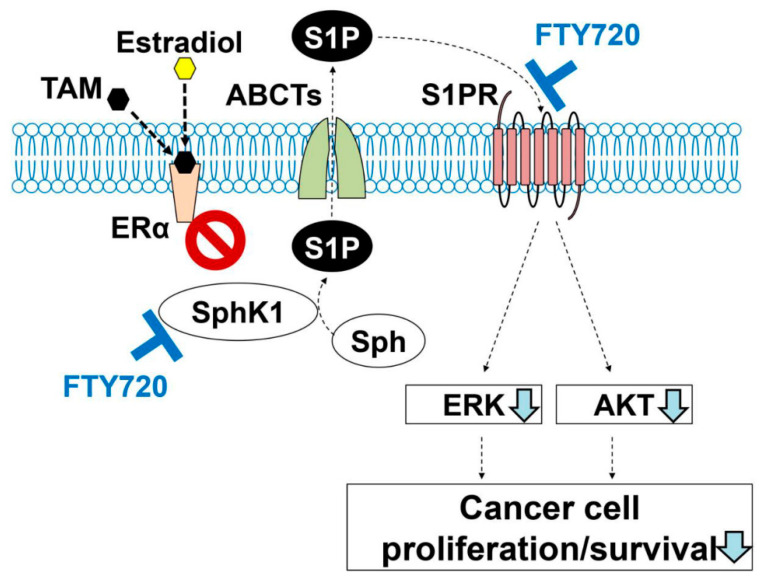
Scheme of blockade of S1P signaling in estrogen receptor (ER)-positive breast cancer (BC) with hormone therapy resistance. In the situation of hormone therapy resistance with higher production and release of S1P by constitutive activation of SphK1, FTY720 can effectively inhibit S1P signaling by blocking both SphK1 and S1PR1.

To test our hypothesis, we next examined the expression levels of S1P-related genes in addition to the ESR1, PGR, and ERBB2 on both wildtype MCF-7 cells (MCF-7/S0.5) and tamoxifen-resistant MCF-7 cells (MCF-7/TAMR-1; Figure 3). ESR1 and ERBB2 have been known to contribute to tamoxifen resistance [44,45,46]. Indeed, we confirmed a significant increase of mRNA of ESR1 and ERBB2 in tamoxifen-resistant MCF-7 cells compared to the wild type (Figure 3). Interestingly, expression of SphK1 as well as S1PR3 were significantly higher in MCF-7/TAMR-1 cells than in MCF-7/S0.5 cells. We next examined the effect of FTY720, a functional antagonist of S1P receptors except S1PR2, which also blocks SphK1 on both cell lines. FTY720 suppressed the viability, of not only MCF-7/S0.5 cells, but also tamoxifen-resistant MCF-7 cells (Figure 4A). Tamoxifen decreased the viability of wildtype MCF-7 cells, but not MCF-7/TAMR-1 cells, as expected (Figure 4B). In contrast, FTY720 successfully decreased not only the viability of MCF-7/S0.5 cells, but also of MCF-7/TAMR-1 cells (Figure 4B). We further examined the involvement of S1PR3 in the viability of MCF-7/S0.5 and MCF-7/TAMR1 cells, using CAY10444, a selective antagonist for S1PR3. CAY10444 slightly decreased the viability of MCF-7/S0.5 cells, and the effect of tamoxifen on MCF-7/S0.5 cell viability was not changed by adding CAY10444 (Figure 4C). Single-agent treatment with tamoxifen or CAY10444 did not significantly suppress MCF-7/TAMR-1 cell viability. However, the combination of tamoxifen and CAY10444 significantly decreased the viability of MCF-7/TAMR-1 cells (Figure 4C). These findings suggest that S1P signaling contributes to cell proliferation and survival in ER-positive BC, and FTY720 appears to be effective in increasing effectiveness of treatment for ER-positive BC, including hormone therapy-resistant types, by blocking the downstream signaling pathway of non-genomic action of estradiol (Figure 5).

## 3. Discussion

Numerous in vitro studies and scarce clinical data have revealed an essential role of the lipid mediator S1P in cancer progression and metastasis, suggesting that targeting S1P signaling may be a promising new strategy for the treatment of cancer [7,47,48,49]. Since S1P is a lipid that is technically difficult to be quantified, the clinical significance of S1P is yet to be established by determining its levels in cancer patients. By applying mass spectrometry, we accurately quantified fine lipid mediators, and measured lipid mediators, not only in basic experiments, but also in clinical specimens, such as tissues and body fluids [15,28,50]. We have previously reported plasma S1P levels in all subtypes of BC, but not the other sphingolipids [51], and comprehensive analyses of systemic plasma levels of sphingolipids in ER-positive BC patients have not been investigated to date. The current study focused on the ER-positive BC, in which S1P plays more important role compared to the other subtypes [28]. Indeed, plasma S1P concentration was found to be associated with breast cancer progression in ER-positive BC patients in the current study, whereas higher histological grade was associated with lower S1P level, most likely reflecting triple-negative BC that has lower levels in our previous study [51]. Moreover, ethnic background of the patients studied were very different between the current and the previous report [51]. The current study investigated Japanese patients, who were 100% Asian, as opposed to the other investigated patients in United States, which was a mixed-ethnicity study [51]. It is known that the Japanese patient population contained more premenopausal patients, who have much more estrogen in the blood compared with the U.S. patients [52]. Since premenopausal patients showed higher levels of S1P, S1P may play a stronger role in this population.

In this study, we demonstrated that plasma S1P levels are higher in patients with larger tumor size, lymph node metastasis, and advanced cancer stage in ER-positive breast cancer patients. Utilizing mass spectrometry, our research group has previously revealed that S1P in cancer tissue from surgical specimens showed significantly higher concentrations compared to S1P in normal breast tissue in BC patients [34]. We further found that higher levels of S1P in cancer tissue are significantly associated with lymph node metastasis in BC patients [35]. Interestingly, we also showed that serum S1P levels gradually increased as tumors grew in mice [15]. Although many findings about S1P and cancer progression have already been reported, as described above, this is the first study to demonstrate that plasma S1P levels are associated with breast cancer progression and its characteristics in patients in ER-positive breast cancer. In the current study, we found a small but significant increase of S1P concentration in the circulation associated with cancer progression, which is in agreement with previous animal experiments [15,19]. We believe that our findings are highly relevant in clinical settings. For instance, higher plasma S1P levels were significantly associated with advanced cancer, such as larger tumor size, lymph node metastasis, and advanced cancer stage. Furthermore, plasma S1P levels are significantly higher in patients with PgR-negative BC than in patients with PgR-positive BC. Given that absence of PgR expression is also related to cancer progression [53], it is suggested that plasma S1P is closely associated with progression of cancer. Moreover, plasma DHS1P levels were significantly higher in patients with higher nuclear grade and pStage. Considering that our previous study showed that not only S1P but also DHS1P is increased in the cancer tissue and associated with cancer progression, it is reasonable to see the higher levels of DHS1P in plasma in patients with more aggressive disease. Taken together, S1P and DHS1P levels not only in the tumor but also in plasma are associated with cancer progression in BC patients. When we measured sphingolipid levels in tumor tissue, we found that not only S1P and DHS1P, but also Sph and DHSph levels were increased in the tumor [34]. To this end, we speculate that aggressive cancer produces S1P by generating its substrates, Sph, and DHSph, which are reflected in plasma.

Our study revealed the clinically meaningful difference of the circulating S1P levels in breast cancer patients by measuring S1P directly, rather than measuring expression levels of SphK1, an S1P-producing enzyme. Few studies have reported the clinical significance of S1P by direct measurement with mass spectrometry, although previous studies indicated a clinical significance of SphK1 measured by RNA or protein expression [20,24,25,35,54]. We believe that the reason why we were able to obtain a clinically meaningful, albeit small, difference of the circulating S1P levels was due to its accurate measurement, which is owed not only to mass spectrometry technology but also appropriate sample handling such as cold storage immediately after blood sample collection. The technology itself may not be new, but it is very difficult to obtain clinically meaningful results, as in this study, even if S1P was measured by methods other than mass spectrometry. We have made various efforts and reported on the experience of S1P measurements in cells, animals, and clinical specimens [15,16,18,26,28,30,50,55]. The accuracy, repeatability, and reproducibility of our mass spectrometry have been repeatedly shown in our previous publications [15,16,28,30,50]. It can be said that S1P measurement by mass spectrometry has already been widespread, but we believe that it is still difficult to obtain absolute values of S1P with consistent and high accuracy from clinical samples partially due to the sample preparation issue in the clinical side.

In the current study, we showed the clinical importance of S1P in progression of ER-positive BC in patients. This finding is consistent with previous experimental data that S1P plays a pivotal role in non-genomic signaling stimulated by estradiol in ER-positive BC [28,40,42]. Recently, it has been reported that SphK1 activation by estrogen receptor α36 contributes to tamoxifen resistance in breast cancer [43]. It was suggested that S1P is critical not only in the progression of ER-positive BC, but also in the development of tamoxifen resistance in those patients [43]. On the basis of these previous findings, we hypothesized that S1P contributes to resistance to hormone therapy and that blocking S1P signaling would improve the susceptibility of hormone therapy-resistant BC (Figure 2D). We tested the hypothesis by in vitro experiments using FTY720 and CAY10444. FTY720 inhibits S1P signaling by blocking S1P receptors, except S1PR2, and suppresses SphK1 [56,57,58,59,60,61]. Moreover, we have previously reported that FTY720 reduces the S1P concentration in the breast cancer cells by the action of phosphorylated FTY720 [62]. Although accumulated data showed the importance of S1P in cancer progression and tamoxifen resistance in ER-positive breast cancer and in mechanisms of how FTY720 works on breast cancer cells, the effect of FTY720 on tamoxifen resistance in breast cancer was never investigated. Therefore, we suppressed S1P signaling by FTY720 in vitro to explore the possibility of a treatment strategy that targets S1P signaling in ER-positive BC patients. In the current study, our findings showed that FTY720 decreased the viability of not only wildtype BC cells, but also of tamoxifen-resistant BC cells. We further examined the involvement of S1PR3 in resistance of BC cells to hormone therapy using CAY10444, a selective antagonist for S1PR3. Although single-agent usage of tamoxifen or CAY10444 did not have a significant suppressive effect on MCF-7/TAMR-1 cells, the combination of tamoxifen and CAY10444 did significantly decrease the viability of MCF-7/TAMR-1 cells. Since FTY720 inhibits both SphK1 and S1PRs, including S1PR3, it appears that the significant effect of FTY720 on tamoxifen-resistant BC cells is partially due to the blocking of S1PR3. Considering these findings, targeting S1P may be a possible new strategy for ER-positive BC patients, including hormone therapy-resistant patients.

One of the major limitations of this study was the retrospective nature of the analysis with a limited number of patients. To our knowledge, however, this is the first study to reveal the association of S1P levels and cancer progression including lymph node metastasis in ER-positive BC patients. We consider this of great interest for the field in that both tissue and plasma S1P levels were associated with cancer progression, as it demonstrates the importance of S1P in actual BC patients. Further clinical studies are warranted to reveal the clinical application of S1P and its related signaling.

In summary, we have determined the concentration of sphingolipids, including S1P, in plasma of patients with ER-positive BC using mass spectrometry. Plasma S1P levels were associated with cancer progression, including lymph node metastasis. Experiments using FTY720 suggested that targeting S1P signaling in ER-positive BC appears to be a possible new strategy, even for hormone therapy-resistant patients.

## 4. Materials and Methods

### 4.1. Plasma Samples from BC Patients

From March 2016 to October 2017, 206 Japanese patients with BC underwent surgical resection in Niigata University Medical and Dental Hospital. During this period, plasma samples were collected from 134 ER-positive BC patients after obtaining informed consent. We analyzed plasma samples from 126 out of the 134 patients, excluding a total of eight patients, including one patient with stage IV, one patient with local recurrence, and six patients with bilateral BC. All plasma samples were collected in tubes after centrifugation, snap-frozen, and stored at −80 °C. This study was approved by the Institutional Review Board of Niigata University.

### 4.2. Quantification of Sphingolipids by Mass Spectrometry

After extraction of lipids from the plasma samples, sphingolipids including S1P were quantified by liquid chromatography, electrospray ionization–tandem mass spectrometry (LC–ESI–MS/MS, 4000 QTRAP, ABI) at the Virginia Commonwealth University Lipidomics Core as described previously [15,28,50]. Internal standards were purchased from Avanti Polar Lipids (Alabaster, AL, USA) and added to samples in 20 μL ethanol/methanol/water (7:2:1) in a cocktail of 500 pmol each. HPLC-grade solvents were obtained from VWR (West Chester, PA, USA).

### 4.3. Comparison of Sphingolipid Levels in Plasma According to Clinicopathologic Characteristics of BC Patients

Histopathological findings were described according to the American Joint Committee on Cancer (AJCC) Staging Manual Eighth Edition [41]. Levels of S1P and other sphingolipids in ER-positive BC patients were analyzed on the basis of clinicopathological factors, including age, menopausal status, body mass index, ER expression, PgR expression, HER2 overexpression/amplification, NSAS nuclear grade, lymphatic invasion, vascular invasion, Ki-67 labeling index, pT category, pN category, and pStage.

### 4.4. Cell Lines and Standard Culture Conditions

The human BC cell lines MCF-7/TAMR-1 and MCF-7/S0.5 were purchased from EMD Millipore. The tamoxifen-resistant cell line MCF-7/TAMR-1 was derived from the MCF-7/S0.5 cell line adapted to grow at low serum concentrations and was established under long-term culture conditions with 1 μM tamoxifen. Cells were cultured in DMEM/F12 medium without phenol red (Sigma Cat. No. D6434), containing 1% FBS (EMD Millipore Cat. No. ES- 009-B), 2.5 mM L-glutamine (EMD Millipore Cat. No. TMS-002-C), and 6 ng/mL insulin (Sigma Cat. No. I-9278) with/without 1 μM tamoxifen (Sigma Cat. No. T5648).

### 4.5. RNA Sequencing

The human BC cell lines MCF-7/TAMR-1 and MCF-7/S0.5 were analyzed by RNA sequencing. Complementary DNAs were prepared from the cell lines with the use of TruSeq Stranded mRNA Library Prep (illumina, Hercules, CA, USA) and sequenced by NovaSeq 6000 (illumina, Hercules, CA, USA). Sequence reads were mapped to the reference genome. The expression level of each transcript was measured as fragments per kilobase of exon per million mapped fragments (FPKM).

### 4.6. Cell Viability Assay

A total of 2000 cells per well were seeded in a 96-well plate. Then, 48 h after pre-culture, the culture media was replaced with drug-containing media at a range of concentrations of FTY720 (0–41.25 μM), 1.5 μM of tamoxifen, or 10 μM of CAY10444 (Cayman Chemical Cat. No. 10005033), or a combination of these drugs. Cells were further incubated for 72 h. Then, cell viability was assessed using the Cell Counting Kit-8 (water-soluble tetrazolium salt; WST-8) assay (Dojindo Cat. No. CK04) following the manufacturer’s instructions. Experiments were set up in sextuplet and repeated three times.

### 4.7. Statistical Analysis

The SPSS 26.0J software package (SPSS Japan, Tokyo, Japan) was used for all statistical evaluations in the study. Categorical variables were compared by Fisher’s exact test or the Pearson χ^2^ test, and continuous variables between two groups were compared by the Mann–Whitney *U* test. All tests were performed as two-tailed tests, and *p*-values < 0.05 were considered statistically significant.

## Figures and Tables

**Table 1:** Clinicopathologic characteristics of 126 BC patients.

Characteristics	Number of Patients (%)
Age (year)	
<60	68 (54%)
≥60	58 (46%)
Menopausal status	
Premenopause	50 (40%)
Postmenopause	75 (59%)
N.D.	1 (1%)
Body mass index	
<25	85 (67%)
≥25	41 (33%)
Estrogen receptor expression	
Negative	0 (0%)
Positive	126 (100%)
Progesterone receptor expression	
Negative	16 (13%)
Positive	110 (87%)
HER2 overexpression/amplification	
Negative	107 (85%)
Positive	12 (10%)
N.D.	7 (6%)
NSAS nuclear grade	
1	73 (58%)
2	24 (19%)
3	27 (21%)
N.D.	2 (2%)
Lymphatic invasion (ly)	
Absent	116 (92%)
Present	10 (8%)
Venous invasion (v)	
Absent	121 (96%)
Present	5 (4%)
Ki-67 labeling index	
<20	77 (61%)
≥20	49 (39%)
Pathological primary tumor (pT) category *	
pTis	19 (15%)
pT1	65 (52%)
pT2	32 (25%)
pT3	9 (7%)
pT4	1 (1%)
Pathological regional lymph node (pN) category *	
pN0	90 (72%)
pN1	23 (18%)
pN2	8 (6%)
pN3	4 (3%)
pNX	1 (1%)
Pathological stage (pStage) *	
pStage 0	19 (15%)
pStage I	50 (39%)
pStage II	41 (33%)
pStage III	15 (12%)
N.D.	1 (1%)

* According to the American Joint Committee on Cancer (AJCC) Cancer Staging Manual Eighth Edition [41]. N.D., not described.

**Table 2:** Levels of DHS1P, Sph, and DHSph in plasma of estrogen receptor-positive breast cancer patients.

		DHS1P (pmol/mL)			Sph (pmol/mL)			DHSph (pmol/mL)		
	No.	Median	(IQR)	*p*	Median	(IQR)	*p*	Median	(IQR)	*p*
Age (year)				0.573			0.200			0.795
60	68	181.5	(52.4)		20.30	(10.0)		14.53	(7.31)	
≥60	58	169.3	(68.3)		18.65	(8.02)		13.74	(6.54)	
Menopause				0.823			0.051			0.774
Premenopause	50	179.7	(57.3)		21.65	(10.59)		14.48	(6.88)	
Postmenopause	75	172.4	(60.5)		18.65	(8.22)		14.00	(6.70)	
Body mass index				0.630			0.409			0.452
25	85	184.6	(62.6)		19.77	(9.47)		13.32	(7.41)	
≥25	41	168.8	(55.4)		18.65	(8.31)		14.83	(6.39)	
PgR expression				0.069			0.369			0.026
Negative	16	201.6	(56.2)		21.83	(9.79)		16.54	(6.92)	
Positive	110	175.5	(59.1)		19.02	(8.74)		13.42	(6.97)	
HER2 expression				0.934			0.196			0.873
Negative	107	182.2	(60.0)		19.07	(8.91)		14.11	(6.70)	
Positive	12	168.5	(69.3)		18.11	(6.93)		12.43	(8.31)	
NSAS nuclear grade				0.029			0.104			0.085
1, 2	97	169.7	(61.4)		18.92	(7.98)		13.36	(7.04)	
3	27	195.5	(49.9)		23.50	(13.1)		15.78	(8.92)	
Lympatic invasion				0.518			0.468			0.281
Negative	116	179.3	(62.8)		19.49	(8.89)		13.74	(7.08)	
Positive	10	189.3	(61.3)		16.66	(8.98)		15.88	(5.06)	
Vascular invasion				0.151			0.942			0.372
Negative	121	182.2	(62.6)		19.23	(8.98)		14.00	(6.78)	
Positive	5	161.6	(28.4)		18.65	(9.44)		15.83	(8.68)	
Ki-67 labeling index				0.568			0.667			0.897
20	77	181.9	(58.4)		19.23	(8.45)		14.46	(6.32)	
≥20	49	176.7	(64.9)		18.86	(12.44)		14.11	(7.46)	
pT				0.066			0.332			0.261
is, 1	85	177.3	(58.4)		18.75	(9.26)		13.48	(7.28)	
2–4	41	193.1	(69.8)		20.19	(7.17)		15.31	(6.83)	
pN				0.347			0.024			0.319
0	90	176.7	(62.0)		18.83	(8.63)		13.34	(8.42)	
1–3	35	184.4	(60.3)		20.83	(10.25)		15.54	(6.64)	
pStage				0.036			0.037			0.241
0, I	69	168.8	(61.5)		18.54	(9.45)		12.84	(7.29)	
II, III	56	186.1	(60.3)		20.45	(7.57)		15.28	(7.25)	

DHS1P, dihydro-sphingosine-1-phosphate; Sph, sphingosine; DHSph, dihydro-sphingosine; IQR, intequartile range; PgR, progesterone receptor; pT, pathological primary tumor; pN, pathological regional lymph node; pStage, pathologiccal stage.

## Supplementary Materials

The following are available online at https://www.mdpi.com/article/10.3390/ijms222413367/s1.

## References

[1] Siegel R.L., Miller K.D., Fuchs H.E., Jemal A. (2021). Cancer Statistics, 2021. CA Cancer J. Clin..

[2] Chaffer C.L., Weinberg R.A. (2011). A perspective on cancer cell metastasis. Science.

[3] Gupta G.P., Massague J. (2006). Cancer metastasis: Building a framework. Cell.

[4] Riggio A.I., Varley K.E., Welm A.L. (2021). The lingering mysteries of metastatic recurrence in breast cancer. Br. J. Cancer.

[5] Spiegel S., Milstien S. (2003). Sphingosine-1-phosphate: An enigmatic signalling lipid. Nat. Rev. Mol. Cell Biol..

[6] Spiegel S., Milstien S. (2011). The outs and the ins of sphingosine-1-phosphate in immunity. Nat. Rev. Immunol..

[7] Ogretmen B., Hannun Y.A. (2004). Biologically active sphingolipids in cancer pathogenesis and treatment. Nat. Rev. Cancer.

[8] Nagahashi M., Takabe K., Terracina K.P., Soma D., Hirose Y., Kobayashi T., Matsuda Y., Wakai T. (2014). Sphingosine-1-phosphate transporters as targets for cancer therapy. BioMed Res. Int..

[9] Moro K., Nagahashi M., Ramanathan R., Takabe K., Wakai T. (2016). Resolvins and omega three polyunsaturated fatty acids: Clinical implications in inflammatory diseases and cancer. World J. Clin. Cases.

[10] Tsuchida J., Nagahashi M., Takabe K., Wakai T. (2017). Clinical Impact of Sphingosine-1-Phosphate in Breast Cancer. Mediat. Inflamm..

[11] Moro K., Kawaguchi T., Tsuchida J., Gabriel E., Qi Q., Yan L., Wakai T., Takabe K., Nagahashi M. (2018). Ceramide species are elevated in human breast cancer and are associated with less aggressiveness. Oncotarget.

[12] Moro K., Nagahashi M., Gabriel E., Takabe K., Wakai T. (2019). Clinical application of ceramide in cancer treatment. Breast Cancer.

[13] Anelli V., Gault C.R., Snider A.J., Obeid L.M. (2010). Role of sphingosine kinase-1 in paracrine/transcellular angiogenesis and lymphangiogenesis in vitro. FASEB J..

[14] Takabe K., Spiegel S. (2014). Export of sphingosine-1-phosphate and cancer progression. J. Lipid Res..

[15] Nagahashi M., Ramachandran S., Kim E.Y., Allegood J.C., Rashid O.M., Yamada A., Zhao R., Milstien S., Zhou H., Spiegel S. (2012). Sphingosine-1-phosphate produced by sphingosine kinase 1 promotes breast cancer progression by stimulating angiogenesis and lymphangiogenesis. Cancer Res..

[16] Liang J., Nagahashi M., Kim E.Y., Harikumar K.B., Yamada A., Huang W.C., Hait N.C., Allegood J.C., Price M.M., Avni D. (2013). Sphingosine-1-phosphate links persistent STAT3 activation, chronic intestinal inflammation, and development of colitis-associated cancer. Cancer Cell.

[17] Nagahashi M., Matsuda Y., Moro K., Tsuchida J., Soma D., Hirose Y., Kobayashi T., Kosugi S., Takabe K., Komatsu M. (2016). DNA damage response and sphingolipid signaling in liver diseases. Surg. Today.

[18] Nagahashi M., Yamada A., Miyazaki H., Allegood J.C., Tsuchida J., Aoyagi T., Huang W.C., Terracina K.P., Adams B.J., Rashid O.M. (2016). Interstitial Fluid Sphingosine-1-Phosphate in Murine Mammary Gland and Cancer and Human Breast Tissue and Cancer Determined by Novel Methods. J. Mammary Gland. Biol. Neoplasia.

[19] Nagahashi M., Yamada A., Katsuta E., Aoyagi T., Huang W.C., Terracina K.P., Hait N.C., Allegood J.C., Tsuchida J., Yuza K. (2018). Targeting the SphK1/S1P/S1PR1 axis that links obesity, chronic inflammation and breast cancer metastasis. Cancer Res..

[20] Hanyu T., Nagahashi M., Ichikawa H., Ishikawa T., Kobayashi T., Wakai T. (2018). Expression of phosphorylated sphingosine kinase 1 is associated with diffuse type and lymphatic invasion in human gastric cancer. Surgery.

[21] Yuza K., Nakajima M., Nagahashi M., Tsuchida J., Hirose Y., Miura K., Tajima Y., Abe M., Sakimura K., Takabe K. (2018). Different Roles of Sphingosine Kinase 1 and 2 in Pancreatic Cancer Progression. J. Surg. Res..

[22] Yuza K., Nagahashi M., Shimada Y., Nakano M., Tajima Y., Kameyama H., Nakajima M., Takabe K., Wakai T. (2018). Upregulation of phosphorylated sphingosine kinase 1 expression in colitis-associated cancer. J. Surg. Res..

[23] Hirose Y., Nagahashi M., Katsuta E., Yuza K., Miura K., Sakata J., Kobayashi T., Ichikawa H., Shimada Y., Kameyama H. (2018). Generation of sphingosine-1-phosphate is enhanced in biliary tract cancer patients and is associated with lymphatic metastasis. Sci. Rep..

[24] Tsuchida J., Nagahashi M., Nakajima M., Katsuta E., Rashid O.M., Qi Q., Yan L., Okuda S., Takabe K., Wakai T. (2020). Sphingosine Kinase 1 is Associated With Immune Cell-Related Gene Expressions in Human Breast Cancer. J. Surg. Res..

[25] Nemoto M., Ichikawa H., Nagahashi M., Hanyu T., Ishikawa T., Kano Y., Muneoka Y., Wakai T. (2019). Phospho-Sphingosine Kinase 1 Expression in Lymphatic Spread of Esophageal Squamous Cell Carcinoma. J. Surg. Res..

[26] Miura K., Nagahashi M., Prasoon P., Hirose Y., Kobayashi T., Sakata J., Abe M., Sakimura K., Matsuda Y., Butash A.L. (2021). Dysregulation of sphingolipid metabolic enzymes leads to high levels of sphingosine-1-phospate and ceramide in human hepatocellular carcinoma. Hepatol. Res. Off. J. Jpn. Soc. Hepatol..

[27] Pitson S.M., Xia P., Leclercq T.M., Moretti P.A., Zebol J.R., Lynn H.E., Wattenberg B.W., Vadas M.A. (2005). Phosphorylation-dependent translocation of sphingosine kinase to the plasma membrane drives its oncogenic signalling. J. Exp. Med..

[28] Takabe K., Kim R.H., Allegood J.C., Mitra P., Ramachandran S., Nagahashi M., Harikumar K.B., Hait N.C., Milstien S., Spiegel S. (2010). Estradiol induces export of sphingosine 1-phosphate from breast cancer cells via ABCC1 and ABCG2. J. Biol. Chem..

[29] Yamada A., Ishikawa T., Ota I., Kimura M., Shimizu D., Tanabe M., Chishima T., Sasaki T., Ichikawa Y., Morita S. (2013). High expression of ATP-binding cassette transporter ABCC11 in breast tumors is associated with aggressive subtypes and low disease-free survival. Breast Cancer Res. Treat..

[30] Nagahashi M., Kim E.Y., Yamada A., Ramachandran S., Allegood J.C., Hait N.C., Maceyka M., Milstien S., Takabe K., Spiegel S. (2013). Spns2, a transporter of phosphorylated sphingoid bases, regulates their blood and lymph levels, and the lymphatic network. FASEB J..

[31] Takabe K., Paugh S.W., Milstien S., Spiegel S. (2008). “Inside-out” signaling of sphingosine-1-phosphate: Therapeutic targets. Pharmacol. Rev..

[32] Matloubian M., Lo C.G., Cinamon G., Lesneski M.J., Xu Y., Brinkmann V., Allende M.L., Proia R.L., Cyster J.G. (2004). Lymphocyte egress from thymus and peripheral lymphoid organs is dependent on S1P receptor 1. Nature.

[33] Pham T.H., Okada T., Matloubian M., Lo C.G., Cyster J.G. (2008). S1P1 receptor signaling overrides retention mediated by G alpha i-coupled receptors to promote T cell egress. Immunity.

[34] Nagahashi M., Tsuchida J., Moro K., Hasegawa M., Tatsuda K., Woelfel I.A., Takabe K., Wakai T. (2016). High levels of sphingolipids in human breast cancer. J. Surg. Res..

[35] Tsuchida J., Nagahashi M., Nakajima M., Moro K., Tatsuda K., Ramanathan R., Takabe K., Wakai T. (2016). Breast cancer sphingosine-1-phosphate is associated with phospho-sphingosine kinase 1 and lymphatic metastasis. J. Surg. Res..

[36] Nava V.E., Hobson J.P., Murthy S., Milstien S., Spiegel S. (2002). Sphingosine kinase type 1 promotes estrogen-dependent tumorigenesis of breast cancer MCF-7 cells. Exp. Cell Res..

[37] Sarkar S., Maceyka M., Hait N.C., Paugh S.W., Sankala H., Milstien S., Spiegel S. (2005). Sphingosine kinase 1 is required for migration, proliferation and survival of MCF-7 human breast cancer cells. FEBS Lett..

[38] Sukocheva O., Wadham C., Holmes A., Albanese N., Verrier E., Feng F., Bernal A., Derian C.K., Ullrich A., Vadas M.A. (2006). Estrogen transactivates EGFR via the sphingosine 1-phosphate receptor Edg-3: The role of sphingosine kinase-1. J. Cell Biol..

[39] Sukocheva O., Wadham C., Xia P. (2013). Estrogen defines the dynamics and destination of transactivated EGF receptor in breast cancer cells: Role of S1P(3) receptor and Cdc42. Exp. Cell Res..

[40] Maczis M., Milstien S., Spiegel S. (2016). Sphingosine-1-phosphate and estrogen signaling in breast cancer. Adv. Biol. Regul..

[41] Amin M.B. (2017). AJCC Cancer Staging Manual.

[42] Watson C., Long J.S., Orange C., Tannahill C.L., Mallon E., McGlynn L.M., Pyne S., Pyne N.J., Edwards J. (2010). High expression of sphingosine 1-phosphate receptors, S1P1 and S1P3, sphingosine kinase 1, and extracellular signal-regulated kinase-1/2 is associated with development of tamoxifen resistance in estrogen receptor-positive breast cancer patients. Am. J. Pathol..

[43] Maczis M.A., Maceyka M., Waters M.R., Newton J., Singh M., Rigsby M.F., Turner T.H., Alzubi M.A., Harrell J.C., Milstien S. (2018). Sphingosine kinase 1 activation by estrogen receptor alpha36 contributes to tamoxifen resistance in breast cancer. J. Lipid Res..

[44] Kim C., Tang G., Pogue-Geile K.L., Costantino J.P., Baehner F.L., Baker J., Cronin M.T., Watson D., Shak S., Bohn O.L. (2011). Estrogen receptor (ESR1) mRNA expression and benefit from tamoxifen in the treatment and prevention of estrogen receptor-positive breast cancer. J. Clin. Oncol..

[45] Dowsett M., Allred C., Knox J., Quinn E., Salter J., Wale C., Cuzick J., Houghton J., Williams N., Mallon E. (2008). Relationship between quantitative estrogen and progesterone receptor expression and human epidermal growth factor receptor 2 (HER-2) status with recurrence in the Arimidex, Tamoxifen, Alone or in Combination trial. J. Clin. Oncol..

[46] De Placido S., De Laurentiis M., Carlomagno C., Gallo C., Perrone F., Pepe S., Ruggiero A., Marinelli A., Pagliarulo C., Panico L. (2003). Twenty-year results of the Naples GUN randomized trial: Predictive factors of adjuvant tamoxifen efficacy in early breast cancer. Clin. Cancer Res..

[47] Pyne N.J., Pyne S. (2010). Sphingosine 1-phosphate and cancer. Nat. Rev. Cancer.

[48] Aoyagi T., Nagahashi M., Yamada A., Takabe K. (2012). The role of sphingosine-1-phosphate in breast cancer tumor-induced lymphangiogenesis. Lymphat. Res. Biol..

[49] Takabe K., Yamada A., Rashid O.M., Adams B.J., Huang W.C., Aoyagi T., Nagahashi M. (2012). Twofer anti-vascular therapy targeting sphingosine-1-phosphate for breast cancer. Gland Surg..

[50] Hait N.C., Allegood J., Maceyka M., Strub G.M., Harikumar K.B., Singh S.K., Luo C., Marmorstein R., Kordula T., Milstien S. (2009). Regulation of histone acetylation in the nucleus by sphingosine-1-phosphate. Science.

[51] Ramanathan R., Raza A., Sturgill J., Lyon D., Young J., Hait N.C., Takabe K. (2017). Paradoxical Association of Postoperative Plasma Sphingosine-1-Phosphate with Breast Cancer Aggressiveness and Chemotherapy. Mediat. Inflamm..

[52] Tsuchida J., Nagahashi M., Rashid O.M., Takabe K., Wakai T. (2015). At what age should screening mammography be recommended for Asian women?. Cancer Med..

[53] Arpino G., Weiss H., Lee A.V., Schiff R., De Placido S., Osborne C.K., Elledge R.M. (2005). Estrogen receptor-positive, progesterone receptor-negative breast cancer: Association with growth factor receptor expression and tamoxifen resistance. J. Natl. Cancer Inst..

[54] Ruckhaberle E., Rody A., Engels K., Gaetje R., von Minckwitz G., Schiffmann S., Grosch S., Geisslinger G., Holtrich U., Karn T. (2008). Microarray analysis of altered sphingolipid metabolism reveals prognostic significance of sphingosine kinase 1 in breast cancer. Breast Cancer Res. Treat..

[55] Nagahashi M., Yamada A., Aoyagi T., Allegood J., Wakai T., Spiegel S., Takabe K. (2016). Sphingosine-1-phosphate in the lymphatic fluid determined by novel methods. Heliyon.

[56] Kharel Y., Lee S., Snyder A.H., Sheasley-O′neill S.L., Morris M.A., Setiady Y., Zhu R., Zigler M.A., Burcin T.L., Ley K. (2005). Sphingosine kinase 2 is required for modulation of lymphocyte traffic by FTY720. J. Biol. Chem..

[57] Sanna M.G., Wang S.K., Gonzalez-Cabrera P.J., Don A., Marsolais D., Matheu M.P., Wei S.H., Parker I., Jo E., Cheng W.C. (2006). Enhancement of capillary leakage and restoration of lymphocyte egress by a chiral S1P1 antagonist in vivo. Nat. Chem. Biol..

[58] Pchejetski D., Bohler T., Brizuela L., Sauer L., Doumerc N., Golzio M., Salunkhe V., Teissie J., Malavaud B., Waxman J. (2010). FTY720 (fingolimod) sensitizes prostate cancer cells to radiotherapy by inhibition of sphingosine kinase-1. Cancer Res..

[59] Tonelli F., Lim K.G., Loveridge C., Long J., Pitson S.M., Tigyi G., Bittman R., Pyne S., Pyne N.J. (2010). FTY720 and (S)-FTY720 vinylphosphonate inhibit sphingosine kinase 1 and promote its proteasomal degradation in human pulmonary artery smooth muscle, breast cancer and androgen-independent prostate cancer cells. Cell Signal..

[60] Lim K.G., Tonelli F., Li Z., Lu X., Bittman R., Pyne S., Pyne N.J. (2011). FTY720 analogues as sphingosine kinase 1 inhibitors: Enzyme inhibition kinetics, allosterism, proteasomal degradation, and actin rearrangement in MCF-7 breast cancer cells. J. Biol. Chem..

[61] Paugh S.W., Payne S.G., Barbour S.E., Milstien S., Spiegel S. (2003). The immunosuppressant FTY720 is phosphorylated by sphingosine kinase type 2. FEBS Lett..

[62] Hait N.C., Avni D., Yamada A., Nagahashi M., Aoyagi T., Aoki H., Dumur C.I., Zelenko Z., Gallagher E.J., Leroith D. (2015). The phosphorylated prodrug FTY720 is a histone deacetylase inhibitor that reactivates ERalpha expression and enhances hormonal therapy for breast cancer. Oncogenesis.

