# Supplementary material for: Plasma Sphingosine-1-Phosphate Levels Are Associated with Progression of Estrogen Receptor-Positive Breast Cancer
Source: Int J Mol Sci. 2021 Dec 13;22(24):13367. doi: 10.3390/ijms222413367 (PMC8703495; doi:10.3390/ijms222413367)
Supplement: Supplementary file 1 [file ijms-22-13367-s001.zip › ijms-1384907-supplement.pdf]

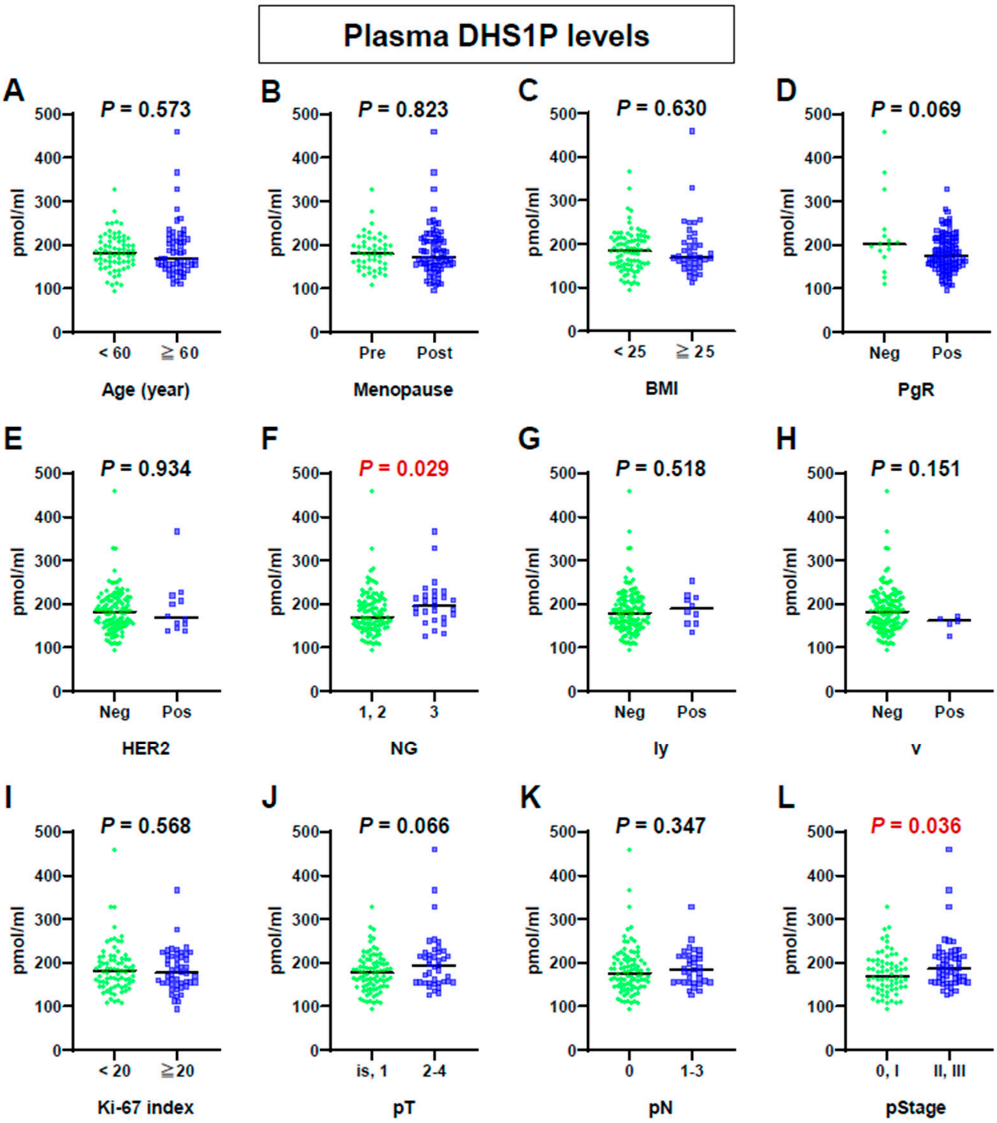

Figure S1: Dihy-dro-sphingosine-1-phosphate (DHS1P) levels in plasma of estrogen receptor-positive breast cancer patients.

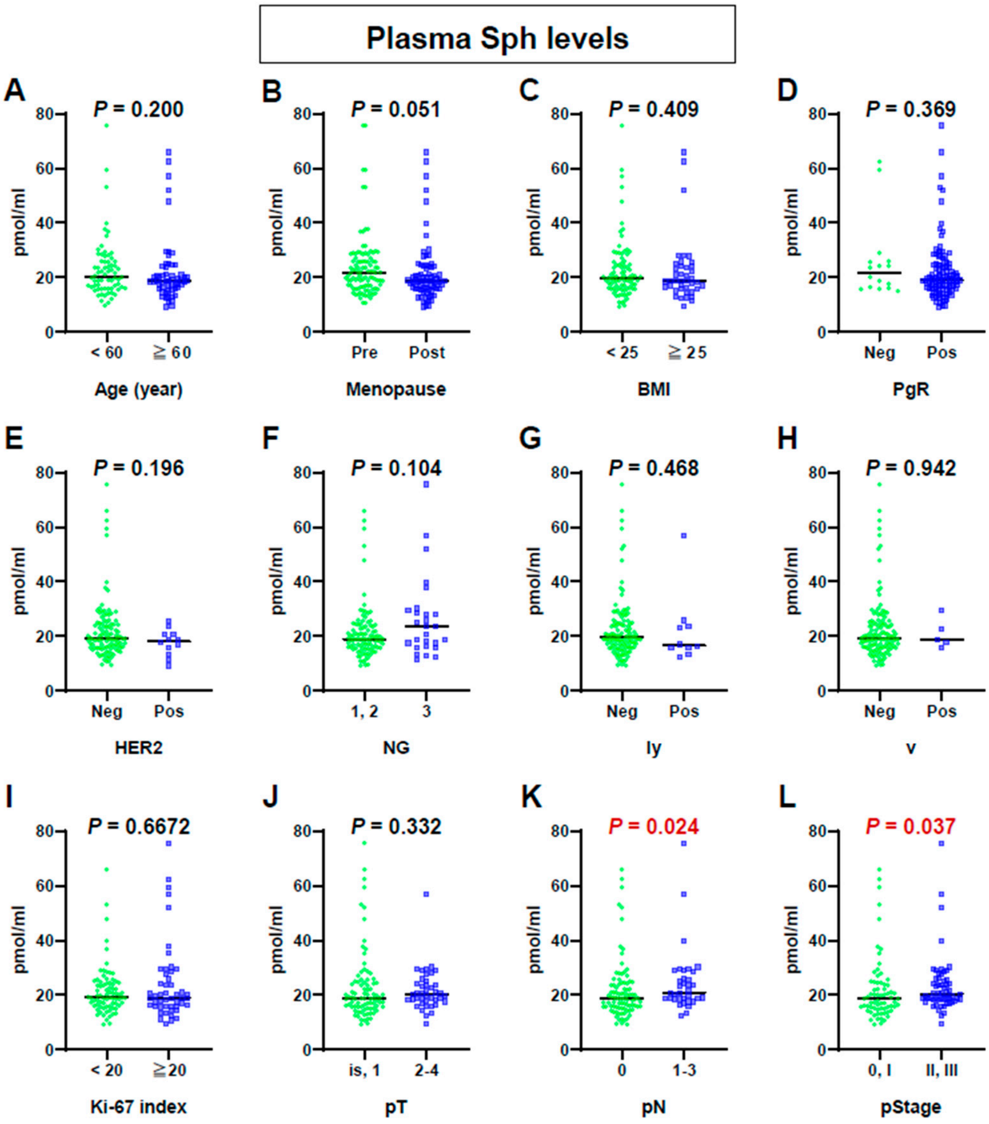

Figure S2: Sphingosine (Sph) levels in plasma of estrogen receptor-positive breast cancer patients.

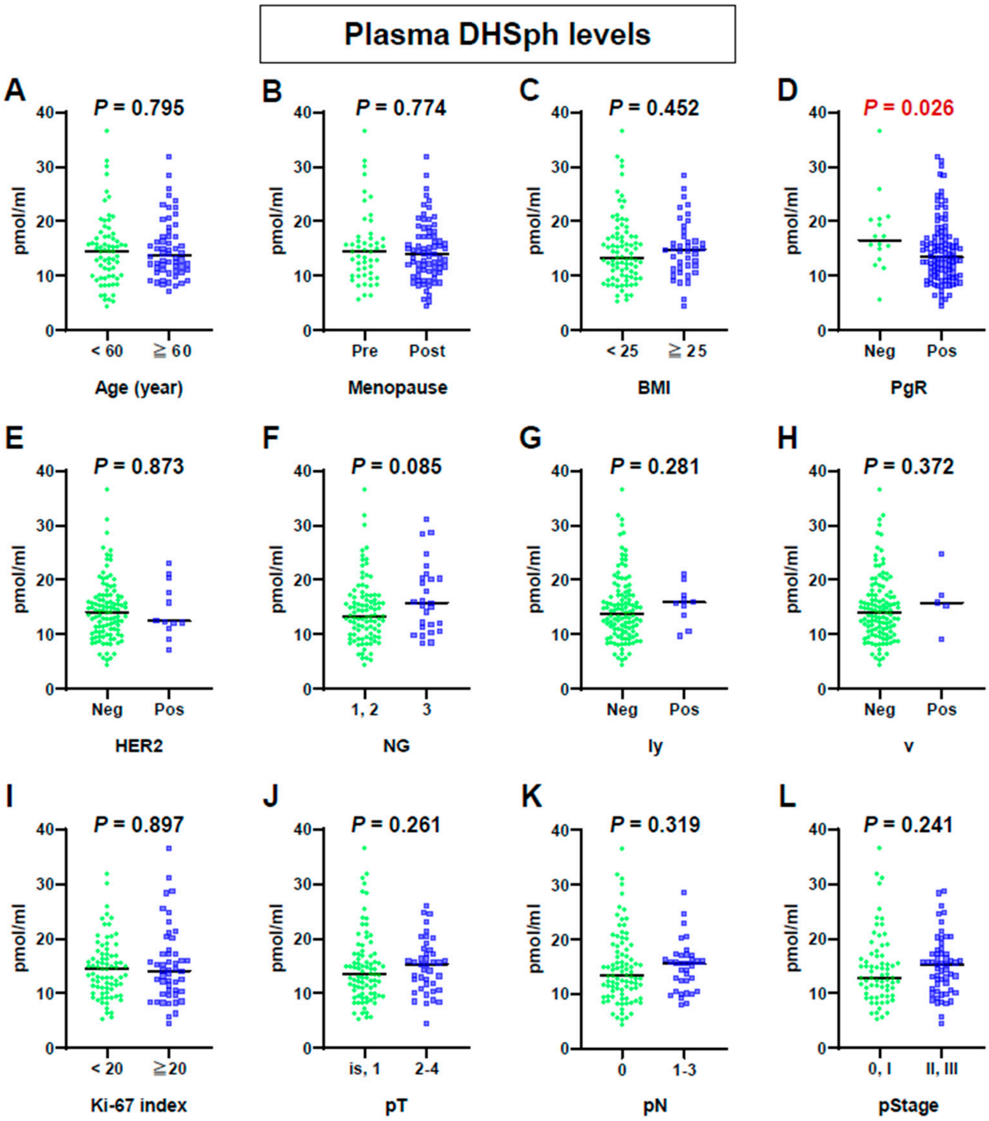

Figure S3: Dihydro-sphingosine (DHSph) levels in plasma of estrogen re-cep-tor-positive breast cancer patients.
